# Low frequency magnetic emissions and resulting induced voltages in a pacemaker by iPod portable music players

**DOI:** 10.1186/1475-925X-7-7

**Published:** 2008-02-01

**Authors:** Howard Bassen

**Affiliations:** 1 U.S. Food and Drug Administration, Silver Spring, MD 20993, USA

## Abstract

**Background:**

Recently, malfunctioning of a cardiac pacemaker electromagnetic, caused by electromagnetic interference (EMI) by fields emitted by personal portable music players was highly publicized around the world. A clinical study of one patient was performed and two types of interference were observed when the clinicians placed a pacemaker programming head and an iPod were placed adjacent to the patient's implanted pacemaker. The authors concluded that "Warning labels may be needed to avoid close contact between pacemakers and iPods". We performed an in-vitro study to evaluate these claims of EMI and present our findings of no-effects" in this paper.

**Methods:**

We performed in-vitro evaluations of the low frequency magnetic field emissions from various models of the Apple Inc. iPod music player. We measured magnetic field emissions with a 3-coil sensor (diameter of 3.5 cm) placed within 1 cm of the surface of the player. Highly localized fields were observed (only existing in a one square cm area). We also measured the voltages induced inside an 'instrumented-can' pacemaker with two standard unipolar leads. Each iPod was placed in the air, 2.7 cm above the pacemaker case. The pacemaker case and leads were placed in a saline filled torso simulator per pacemaker electromagnetic compatibility standard ANSI/AAMI PC69:2000. Voltages inside the can were measured.

**Results:**

Emissions were strongest (≈ 0.2 μT pp) near a few localized points on the cases of the two iPods with hard drives. Emissions consisted of 100 kHz sinusoidal signal with lower frequency (20 msec wide) pulsed amplitude modulation. Voltages induced in the iPods were below the noise level of our instruments (0.5 mV pp in the 0 – 1 kHz band or 2 mV pp in the 0 – 5 MHz bandwidth.

**Conclusion:**

Our measurements of the magnitude and the spatial distribution of low frequency magnetic flux density emissions by 4 different models of iPod portable music players. Levels of less than 0.2 μT exist very close (1 cm) from the case. The measured voltages induced inside an 'instrumented-can' pacemaker were below the noise level of our instruments. Based on the observations of our in-vitro study we conclude that no interference effects can occur in pacemakers exposed to the iPod devices we tested.

## Introduction

Recently, malfunctioning of a cardiac pacemaker electromagnetic, caused by electromagnetic interference (EMI) by fields emitted by personal portable music players, was highly publicized around the world. This media activity was based on a single case report [1]. The report involved a patient who experience syncope that resulted in fall and head injury prior to examination of suspected (EMI). Upon recovery the patient was examined in a clinical setting and the authors suspected an iPod might have caused interference with the pacemaker's operation. An iPod (model not specified) was placed 2 inches above the pacemaker programming head. The programming head (position not specified) was placed adjacent to the patient's implanted pacemaker. One can assume the programmer head was in contact with the skin adjacent to the implanted pacemaker. Two types of interference were observed, oversensing in both atrial and ventricular channels. The pacemaker detected high ventricular rates. The authors concluded that "Warning labels may be needed to avoid close contact between pacemakers and iPods." Soon after, a clinical study was performed and found "no interference for any of the eight pacemakers or the ICD (implantable cardiac defibriilator) [2]."

Our paper describes an in-vitro study of EM fields and the resulting voltages induced in the leads and case of a specially instrumented pacemaker. Data from our study can provide physical information to prove whether it is possible to induce electromagnetic interference in pacemakers. Following this incident FDA's Electromagnetics (EM) and Wireless laboratories performed in-vitro evaluations of various models of the Apple Inc iPod music player for low frequency magnetic field emissions within 5–10 mm of the surface of the player and for voltages induced in an instrumented pacemaker with leads. The instrumented pacemaker was in a saline bath 2.3 cm from the player which was immediately above the bath. Measurements involved 4 iPods (two with hard drives using magnetic coils). Hard drives which can emit magnetic fields depending on their metallic case and other magnetic shielding). The iPod units were tested while playing music at high volume with the earphones connected. The iPod charging wires were not connected.

## Methods

### 1. Emission measurements

We tested four models of iPods, an iPod Nano (second generation), an iPod shuffle (second generation), an iPod Video (fifth generation) with 30 GB hard drive, and a standard iPod (with 15 GB hard drive and dock connector). The specific models wee verified by using the Apple Inc. website [3]. We measured magnetic fields with an ERM model 1987.001 three-axis magnetic field probe and ERM model 1987.002 readout. The probe has a small sensor volume (1 cubic cm) and a frequency response that is constant from 3 kHz to 3 MHz. We scanned each of four iPod models at distances of 5 mm and 10 mm above their front and back surfaces. Data were captured by a digital oscilloscope (WaveRunner model 2, LeCroy Corporation,) at both 500 Mega samples/sec and 500 kilo samples/sec. We saw nothing above the noise level of this probe (0.2 μT = 0.16 A/m = 2 mG). We also looked with a low pass filter from 3 kHz – 100 kHz but saw no magnetic fields above the lower noise level of this narrowband arrangement.

We then used a larger custom built 3-coil sensor by Integrity Research, (Buffalo N.Y. 14222, USA). The coils of the Integrity 3-axis probe are inside a sphere about 3.5 cm in diameter. We calibrated this probe at 100 kHz and other frequencies using the measured field from a 60 cm diameter calibration coil with 30 turns of wire wound on the circumference of a 1 cm thick plastic disc. The calibration was based on the magnetic flux density measured at a point that was quantified using two measurement probe systems (ERM model 1987 (Electric Research and Management, Inc Cabot PA, USA) and a Wandel & Goltermann model EFA-2 with B2116 probe (Narda Safety Test Solutions GmbH, Pfullingen, Germany). The two other probe systems were each calibrated by their manufacturer. The Integrity probe that we used for our actual measurements of iPod emissions has an output voltage that is directly proportional to frequency as well as magnetic flux density. Therefore the Integrity instrument is limited in its ability to observe lower frequencies (e.g. below 10 kHz). The coils of this 3 axis passive probe are inside a sphere about 3.5 cm in diameter. Fields were seen at some locations. Low frequencies fields down to 0 Hz were capable of being observed using other methods described below, but none were seen.

### 2. Induced voltage measurements

**Figure 1:**
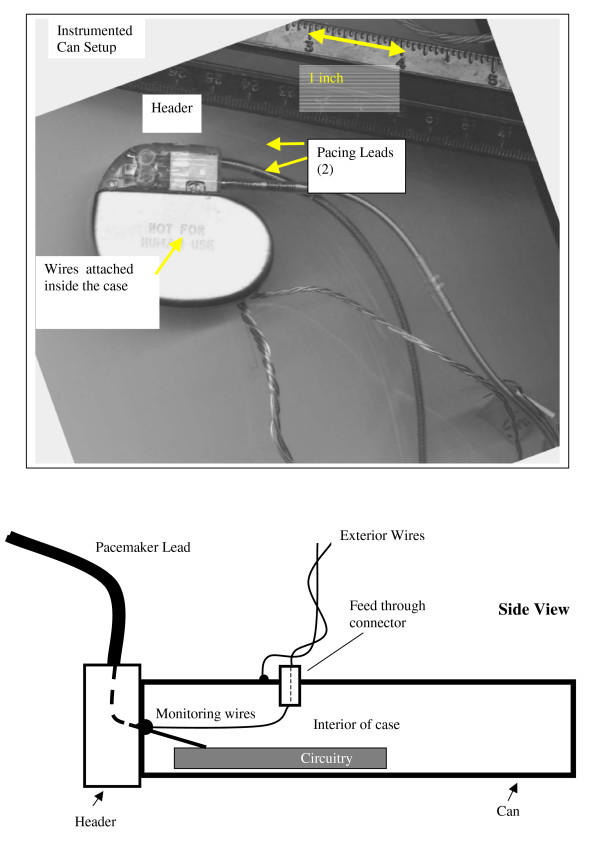
Instrumented Can Pacemaker Setup for Induced Voltages.

**Figure 2:**
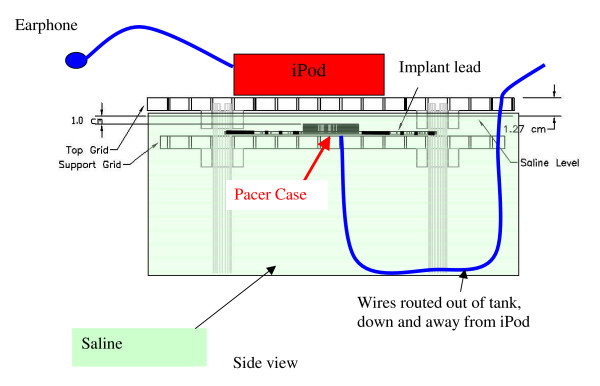
Saline filled torso simulator.

We measured the voltages induced inside a custom made 'instrumented-can' pacemaker (Figure 1). The pacemaker was placed in a saline filled torso simulator (Fig. 2) for testing in accordance with the AAMI PC69 pacemaker EMC standard [4]. This pacemaker had two standard unipolar leads connected to its header. The instrumented can is an actual pacemaker case (can) but with no battery inside the case. A set of insulated wires is attached inside the case to the point where the pacemaker lead enters the can (header connections). These insulated "monitoring wires" allow observation of the voltages that are delivered to the actual pacemaker circuitry. On the outside of the case, insulated wires are connected to the feed through connectors, thus making an electrical connection to the monitoring wires. These exterior wires are approximately 60 cm long cm long. These wires passed through the saline in the torso simulator and continue on, outside of the simulator, to a preamplifier and measuring equipment (Figure 2). The wires were arranged as twisted pairs in a single bundle to minimize induced voltage pickup from the magnetic field.

This measurement technique provided a clear view of the actual voltages that were induced (by the iPods) in a real pacemaker with leads (from approximately 0 to several MHz). Testing was performed in 0.18% saline in the torso simulator. The two pacemaker leads were arranged in loops on the top surface of the torso simulator, submerged in saline. Voltages between the tip and case ("can") and between the lead electrode "ring" and can were measured for each lead. Confirmation of proper operation of the test system was performed by placing the broad surface of the 60-cm diameter calibration coil on top grid of the PC69 test setup. Various AC signals were fed to the coil and voltages were observed on the wires attached to each of the pacemaker leads. After testing the system, each iPod was placed on the top grid of the torso simulator and moved over the entire loop area below the grid. We monitored voltages with an EG&G model 5113 low noise differential amplifier (gain = 1000, 0–1 kHz bandwidth) that drove the input of a digital sampling oscilloscope.

## Results

**Figure 3:**
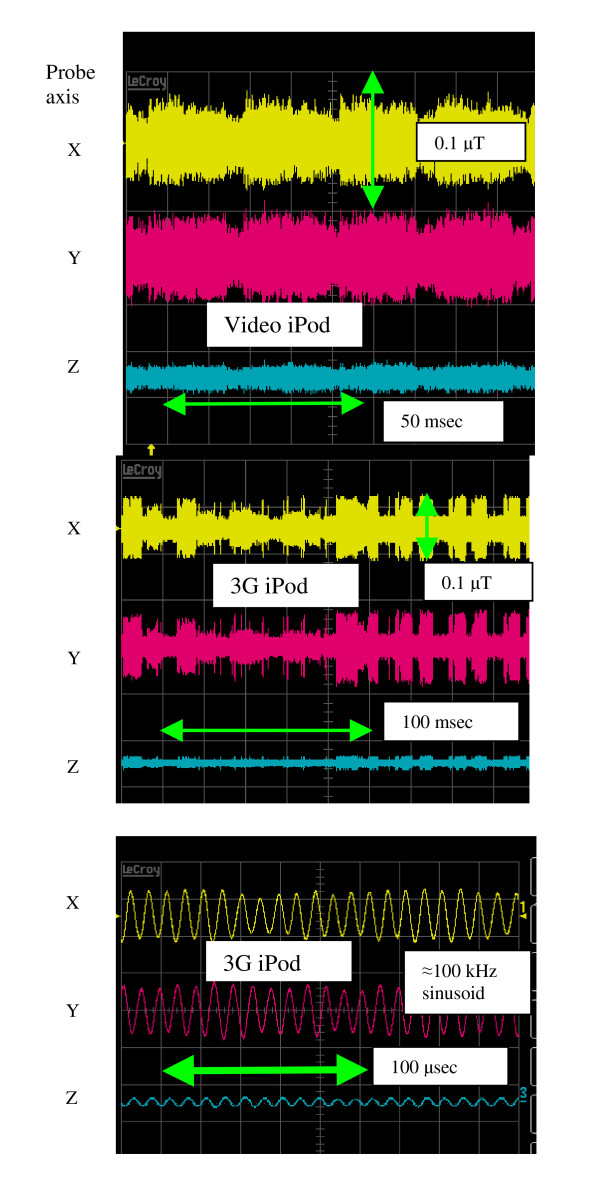
Measured data for magnetic field emissions from the three sensors of the instrument (X, Y and Z axes).

Results for the measurements of flux density with the Integrity instrument are shown in Figure 3. The 3 traces in each figure are the X, Y and Z vector components of the time-domain magnetic flux density. The total field at any one instant is the square root of the sum of the squares of the 3 voltages. Data taken at 0.5 and 1 cm from the case of the iPods indicates a 0.2 μT or less total field with 100 kHz sinusoidal signal that is highly localized. The fields exist only in an area of about one square cm. The field falls off dramatically at distances greater than 1 cm and was not measurable. Fields were strongest close to the case of the two iPods with hard drives. At some locations, the video iPod emissions consisted of with lower frequency (20 msec wide) pulsed amplitude modulation. No measurable fields were found near the earphone cable, except at the earphone itself.

The induced voltage measurements with the Instrumented Can pacemaker revealed nothing above the noise level of 0.5 mV pp (0 – 1 kHz). With the preamp removed to expand the bandwidth to 5 MHz we saw nothing above the noise level of 2 mV pp.

Data measured by another group [5] revealed information about the voltage induced in a 6 cm diameter loop antenna over the range of 50 Hz to 900 MHz. The induced voltage was 71 microVolts into a 50 ohm load at 56.5 MHz for the 'video iPod. For a higher impedance load the voltage could be twice as much, or 142 microVolts. A pulse repetition frequency of about 30 MHz was identified in the spectral measurements of the fields emitted by the video iPod.

## Discussion

### 1. Emissions

Our Integrity Instruments probe averaged the fields we measured over a 3.5 cm sphere. This is a large volume compared to the volume containing the total field that is emitted by each of the iPods. Due to this spatial averaging of by our probe, we could not determine the true magnetic flux density at any point with reasonable accuracy. In addition our probe was not sensitive to fields below 3 kHz. Both of these limitations were resolved by performing the induced voltage measurements. In addition, the theory of the induced voltage measurements is discussed immediately.

### 2. Induced voltage measurements

The low frequency voltages that could be induced in the pacemaker's leads and inside the case of the pacemaker are obey classical electromagnetic field theory (Faraday's Law) described by equation 1.

$$V_{b}=\oint E•dl=\frac{d}{dt}\int B•dA$$

Where:

V_B _= voltage induced between the can and lead tip

E = electric field

dl = unit element of a closed loop

d/dt = the rate of change with respect to time

B = magnetic flux density

dA = unit element of the surface area A

**Figure 4:**
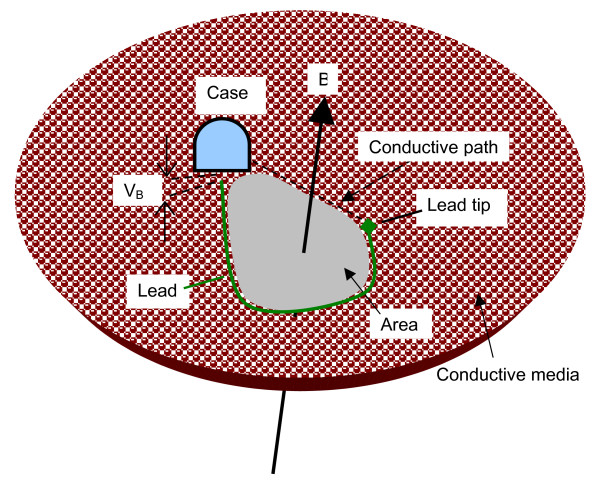
Induced field concept diagram.

When an implanted device with a unipolar lead is exposed to magnetic flux density (B), a voltage (V_B_) is induced in the gap between the case (can) and the distal tip of the lead as shown in Figure 4. This voltage V_B _is defined by the first integral in equation 1 (∮ *E *• *dl*). The integral requires a closed loop (linear path) that is formed by the pacemaker lead (dotted line) plus the conductive path in the saline (see Fig. 4). The path goes between the distal tip of the lead and the case.

Voltage V_B _is also defined by the second integral of equation (1): ($\frac{d}{dt}\int B•dA$). The integral of B dA represents the entire "number" of magnetic flux lines within the closed loop. The term involving the rate of change d/dt in implies that higher frequencies induce proportionally higher voltages in the loop. An important note is that *B *• *dA *indicates that the magnetically induced voltage (V_b_) is at its maximum when the B vector is perpendicular to the area, and B is uniform in strength and direction over the entire loop. For pacemakers with bipolar leads, a much smaller voltage is induced than for pacemakers with unipolar leads since the loop is non-existent – i.e. the there are two distal tips and no sensing is performed via the tips through the conductive path to the can.

Equation (1) allows us to understand why no induced voltage (V_B_) was seen for measurements made with our instrumented can with a pacemaker lead surrounding each of the iPods. This is due to the weak magnitude of the measured flux density B (less than 0.2 μT) and the very small area (a few square centimeters) where any significant flux density exists.

## Conclusion

We performed measurements of the magnitude and the spatial distribution of low frequency magnetic flux density very close to the entire surface of 4 different models of iPod portable music players. Further measurements were made with an actual pacemaker case and leads, in a saline filled torso simulator. The results imply that virtually no low frequency voltages can be induced into the case of an implanted pacemaker or defibrillator through the loop formed by the leads, case, and intervening body tissues. Induced voltages inside the case were undetectable (less than 0.2 mV pp in a 0 – 1 kHz band or less than 2 mV pp in a 0 – 5 MHz band). Data from others indicates voltages of no more than 140 microVolts RMS could be induced in the case and electronics of pacemakers or implanted cardiac defibrillators (ICDs) in the range of 50 Hz to 900 MHz. Based on the miniscule voltages observed in our in-vitro study and others, we conclude that no interference effects can occur in pacemakers exposed to the iPod devices we tested.

Finally, existing EMC standards for active cardiac implants require these devices to be immune to interference from very high intensity (150 A/m) magnetic field strengths [6] frequencies that we evaluated. Our data indicate that all iPods we studied emit very weak fields. Based on these data it is again concluded that it is not possible for interference to be induced in a pacemaker by the music players we tested.
